# MICAL2 implies immunosuppressive features and acts as an independent and adverse prognostic biomarker in pancreatic cancer

**DOI:** 10.1038/s41598-024-52729-6

**Published:** 2024-02-07

**Authors:** Zhicheng Liu, Bing Sun, Aiguo Xu, Jingjiao Tang, Huiqin Zhang, Jie Gao, Lei Wang

**Affiliations:** 1 Department of Oncology, The Affiliated Lianyungang Hospital of Xuzhou Medical University (The First People′s Hospital of Lianyungang), Lianyungang, Jiangsu China; 2 Jinzhou Medical University Postgraduate Training Base (The First People′s Hospital of Lianyungang), Lianyungang, Jiangsu China; 3 Department of Oncology, The Second People’s Hospital of Lianyungang, Lianyungang, Jiangsu China

**Keywords:** Tumour biomarkers, Data integration

## Abstract

At present, clinical outcomes of pancreatic cancer patients are still poor. New therapeutic targets for pancreatic cancer are urgently needed. Previous studies have indicated that Microtubule Associated Monooxygenase, Calponin and LIM Domain Containing 2 (MICAL2) is highly expressed in many tumors and promotes tumor progression. However, the role played by MICAL2 in pancreatic cancer remains unclear. Based on gene expression and clinical information from multiple datasets, we used comprehensive bioinformatics analysis in combination with tissue microarray to explore the function and clinical value of MICAL2. The results showed that MICAL2 was highly expressed in pancreatic cancer tissue and exhibited potential diagnostic capability. High expression of MICAL2 was also associated with poor prognosis and acted as an independent prognostic factor. MICAL2, mainly expressed in fibroblasts of pancreatic cancer, was closely related to metastasis and immune-related features, such as epithelial-mesenchymal transformation, extracellular cell matrix degradation, and inflammatory response. Furthermore, higher MICAL2 expression in pancreatic cancer was also associated with an increase in cancer-associated fibroblasts as well as M2 macrophage infiltration, and a reduction in CD8 + T cell infiltration, thereby facilitating the formation of an immunosuppressive microenvironment. Our results helped elucidate the clinical value and function in metastasis and immunity of MICAL2 in pancreatic cancer. These findings provided potential clinical strategies for diagnosis, targeted therapy combination immunotherapy, and prognosis in patients with pancreatic cancer.

## Introduction

Pancreatic cancer is a highly malignant tumor of the digestive tract, which is difficult to treat and seriously endangers human life and health. In 2020, 495, 773 new cases of pancreatic cancer and 466, 003 deaths were reported worldwide, with a mortality rate that equaled the morbidity rate and a 5-year survival rate of only 9^1,2^. Reportedly, patients with early resectable pancreatic cancer have a maximum survival of 54.4 months following surgical treatment and postoperative adjuvant chemotherapy^3^. However, due to the lack of effective early screening tools and specific clinical features^4^, most diagnosed patients are in the stage of inoperable locally advanced or distant metastasis^5,6^. In addition, owing to the special anatomical location of the pancreas compounded by the insensitivity of pancreatic cancer cells to radiation, radiotherapy doesn't work well. Although some targeted therapeutic agents exert significant therapeutic effects, their efficacy is limited by the low mutation frequency in cancer cell populations, thus only benefiting some patients^7–9^. The fact that immunotherapy, which has seen rapid progress in recent years, has been unable to achieve satisfactory results against pancreatic cancer is mainly due to the nature of connective tissue proliferation and low immune infiltration in the microenvironment of pancreatic cancer^10^.

In particular, studies have shown that the failure of immunotherapy to realize its full potential is mainly attributable to cancer-associated fibroblasts (CAFs), the most abundant cell type in the tumor microenvironment (TME)^11^. CAFs, a stromal cell population with a cellular origin and phenotypic and functional heterogeneity, are the most important components of the TME^12^. CAFs upregulate and release vascular endothelial growth factor (VEGF) and interleukin-6 (IL-6) to promote angiogenesis in tumor tissues^13^. CAFs secrete enzymes required for matrix cross-linking, which promote extracellular matrix (ECM) remodeling. A dense ECM may hinder drug delivery, leading to the poor accumulation of chemotherapeutic agents in tumors, and subsequent therapeutic failure^14^. CAFs also exert immune-suppressive effects via the secretion or up-regulation of IL-6, C-X-C motif chemokine ligand 9 (CXCL9), and transforming growth factor-β (TGF-β), which inhibit the differentiation or infiltration of CD8 + T cells and macrophages^15,16^. Thus, the need to explore new CAFs biomarkers has been urgently felt.

Microtubule Associated Monooxygenase, Calponin and LIM Domain Containing 2 (MICAL2) is a member of the MICAL family and contains several discrete structural domains, including a CH structural domain, LIM structural domain, and a proline-rich motif^17^. The MICAL family plays a key role in the regulation of cytoskeletal dynamics and associated biological processes^18^. In recent years, MICAL2 has been found to be highly expressed in many tumors and promote tumor progression by inducing tumor cell proliferation and migration^19–22^. In gastric cancer, MICAL2 has been shown to promote gastric cell migration by disrupting the impaired binding of E-cadherin to β-catenin, resulting in the accumulation of β-catenin in the nucleus, which enhances β-catenin signaling^23^. High expression of MICAL2 is associated with poor prognosis for glioblastoma patients, as well as the promotion of glioblastoma in mice^24^. However, the biological function of tumorigenesis and the prognostic impact of MICAL2 in pancreatic cancer remains unclear. In this study, we explored the expression level of MICAL2 in pancreatic cancer and normal tissues and evaluated the diagnostic and prognostic capability of MICAL2. Further, we revealed the potential metastasis and immunity function of MICAL2 in public datasets and internal cohort. Our study might provide novel insights into diagnosis, therapy, and prognosis for pancreatic cancer.

## Materials and methods

### Data access and samples

Pancreatic cancer datasets, including high-throughput sequencing data, single-cell sequencing data, and microarray data, were obtained from the Gene Expression Omnibus (GEO) database (https://www.ncbi.nlm.nih.gov/geo/) and The Cancer Genome Atlas (TCGA) database (https://portal.gdc.cancer.gov/). Microarray datasets (GSE16515, GSE183795) were downloaded from the GEO database^25–28^. GSE16515 was processed with RMA as normalization method. GSE183795 incorporated earlier submitted data sets, which were batch-corrected and normalized. GSE16515 contained 16 pairs of normal and pancreatic cancer tissues and an additional 20 pancreatic cancer tissues. GSE183795 contained 45 pairs of normal and pancreatic cancer tissues and an additional 94 pancreatic cancer tissues. The RNA sequencing data (transcripts per million format) and clinical information of 178 pancreatic cancer patients were downloaded from the TCGA database. A total of 184 pancreatic cancer patients in our cohort were collected for tissue microarray. The detailed patient information is shown in Supplementary Table S1.

### Expression and diagnostic capability of MICAL2

Both paired and unpaired statistical analyses were performed for normal and tumor tissues in GSE16515 and GSE183795, and an unpaired analysis for the TCGA and GTEx datasets. To evaluate the relevance of MICAL2 to the diagnosis of pancreatic cancer patients, we conducted receiver operating characteristic (ROC) curves of the GSE183795 and GSE16515 datasets using the "pROC" package^29^.

### Survival analysis and independent prognostic analysis

For survival analysis, we used the "survival" package to analyze the difference in overall survival time and plotted the survival curves according to MICAL2 expression. In TCGA and GSE183795, we divided high and low expression groups according to the median of gene expression. The values of gene expression below the median were classified as the low-expression group, while those above the median were classified as the high-expression group. Next, the univariable and multivariable Cox regression analyses were performed to assess whether MICAL2 is an independent prognosis factor compared to other clinical factors.

### Functional enrichment analysis and single-sample gene set enrichment analysis

First, we screened genes for significantly correlated with MICAL2 expression based on a correlation coefficient > 0.6 and a p-value < 0.05. Next, the Gene Ontology (GO) and Kyoto Encyclopedia of Genes and Genomes (KEGG) enrichment analysis of these genes was analyzed using the DAVID database (https://david.ncifcrf.gov/)^30,31^, following which functional bubble maps were drawn for presentation. We downloaded three gene sets from the GSEA database (https://www.gseamsigdb.org/gsea/index.jsp) as follows: the HALLMARK EPITHELIAL MESENCHYMAL TRANSITION; HALLMARK INFLAMMATORY RESPONSE; and HALLMARK HYPOXIA^32^. Subsequently, we used the "GSVA" package^33^ to score the three above-mentioned gene sets in pancreatic cancer data and analyzed the correlation between MICAL2 and the scores of these three gene sets.

### The assessment of immune score, stromal score, and tumor purity

In the TCGA dataset, we used the "estimate" package to calculate the immune score, representing the level of immune cell enrichment, and the stromal score, representing the stromal cell content. We compared the differences between the immune and stromal scores of patients with high/low MICAL2 expression.

### Single-cell sequencing data analysis

Tumor Immune Single-cell Hub 2 is a single-cell sequencing analysis database that is used to explore the TME^34^. We used the TISCH2 tool to analyze the single-cell sequencing dataset GSE162708 from the GEO database^35^. We focused on the individual cell classification types of this dataset as well as the expression and distribution of MICAL2 in different cell types.

### Tissue microarray and immunohistochemistry staining

Paraffin-embedded 184 pancreatic cancer samples were used to make tissue microarray. The sections were first dewaxed and hydrated in buffer and then performed antigenic thermal repair. After, the sections were blocked endogenous peroxidase activity with ethanol containing 3% hydrogen peroxidase, and incubated overnight with the primary antibody [antibody: CD163, dilution: 1:7200; antibody: Fibroblast Activation Protein Alpha (FAP), dilution: 1:1600; antibody: Actin Alpha 2 (ACTA2), dilution: 1:200; antibody: Matrix Metalloproteinase 2 (MMP2), dilution: 1:2400; antibody: MICAL2, dilution: 1:900] at 4 °C, followed with appropriate secondary antibodies. Finally, the sections were re-stained with hematoxylin, and dehydrated using an alcohol gradient, following which the slides were sealed with a neutral dendrimer. According to the intensity and extent of positive cell expression, quantitative interpretation was made in an immunohistochemistry experiment. The staining intensity reflected the percentage of positive cells: 0 (< 5%), 1 (6–25%), 2 (26–50%), 3 (51–75%), and 4 (> 75%). Grades 0, 1, and 2 were divided into the low-expression group, and the other two grades were the high-expression group. Based on the above grouping, we performed differential analysis and survival analysis.

### The assessment of immune cell infiltration

First, the “CIBERSORT” algorithm was used to obtain data on infiltration by 22 types of immune cells in each patient with pancreatic cancer via the TCGA dataset. We then grouped the two groups according to high/low MICAL2 expression levels to compare the differences in immune cell infiltration of the two groups.

### Statistical analysis

Statistical analysis and graphing were performed using R 4.1.0 and GraphPad Prism 8 software. The data were analyzed for normality first. If the data were not normally distributed, paired samples were statistically analyzed using the wilcoxon test. A Mann–Whitney U test was applied for unpaired samples. Data were shown as either the median with interquartile range or the mean ± standard error of the mean. p < 0.05 was regarded as statistically significant.

### Ethics approval and consent to participate

The study involving humans was approved by the Institutional Review Board of The First People's Hospital of Lianyungang (KY-20200923001-01). The study was conducted by the local legislation and institutional requirements. The participants provided their written informed consent to participate in this study.

## Results

### MICAL2 is highly expressed in pancreatic cancer tissues and has a potential diagnostic capability

**Figure 1:**
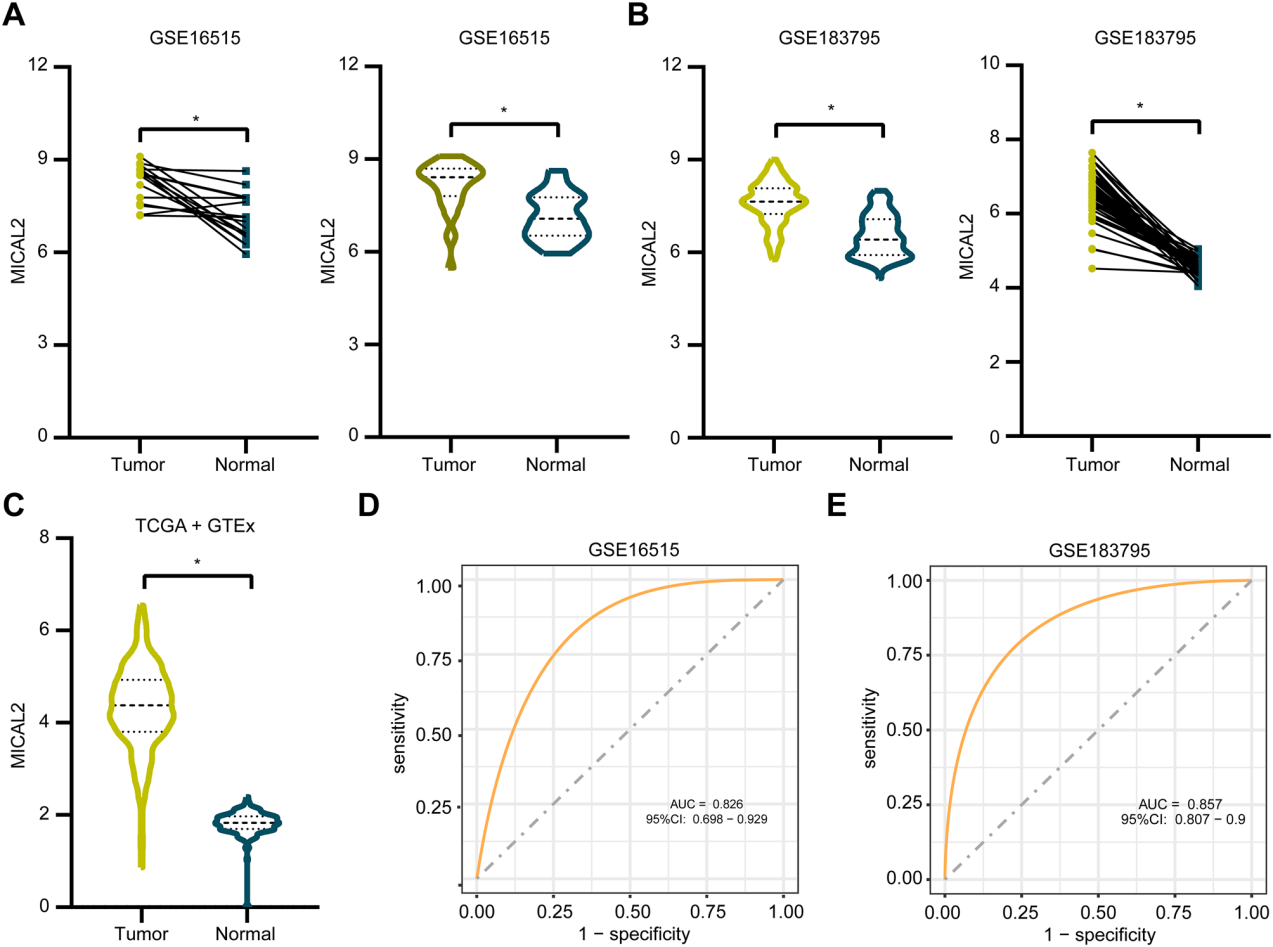
Expression and diagnostic assessment of MICAL2 in pancreatic cancer and normal samples. (**A**) Paired difference analysis (Tumor: 16; Normal: 16) and unpaired difference analysis (Tumor: 36; Normal: 16) of MICAL2 in GSE16515. (**B**) Paired difference analysis (Tumor: 45; Normal: 45) and unpaired difference analysis (Tumor: 139; Normal: 102) of MICAL2 in GSE183795. (**C**) Unpaired difference analysis (Tumor: 178; Normal: 167) of MICAL2 in the TCGA and GTEx dataset. (**D**,**E**) The area under the ROC curve shows the accuracy of MICAL2 expression in the diagnosis of pancreatic cancer in GSE16515 and GSE183795 (*p < 0.05).

We used public datasets on pancreatic cancer to analyze the differential expression of MICAL2. First, the two datasets, GSE16515 and GSE183795, in the GEO database were subjected to paired and unpaired analysis. As shown in Fig. 1A,B, the results indicated that MIACL2 expression in the pancreatic cancer group tissues was higher than that in normal group tissues (p < 0.05). A similar result was obtained by differential analysis of the TCGA and GTEx datasets (p < 0.05, Fig. 1C). To further explore the potential diagnostic value of MICAL2, we performed ROC curve analysis based on GSE183795 and GSE16515 datasets. ROC curves revealed that area under the curves of GSE16515 (Fig. 1D) and GSE183795 (Fig. 1E) were 0.826 [95% confidence interval (CI) 0.698–0.929] and 0.857 (95% CI 0.807–0.9), indicating that MICAL2 shows potential as a diagnostic biomarker for pancreatic cancer.

### The underlying biological function of MICAL2

**Figure 2:**
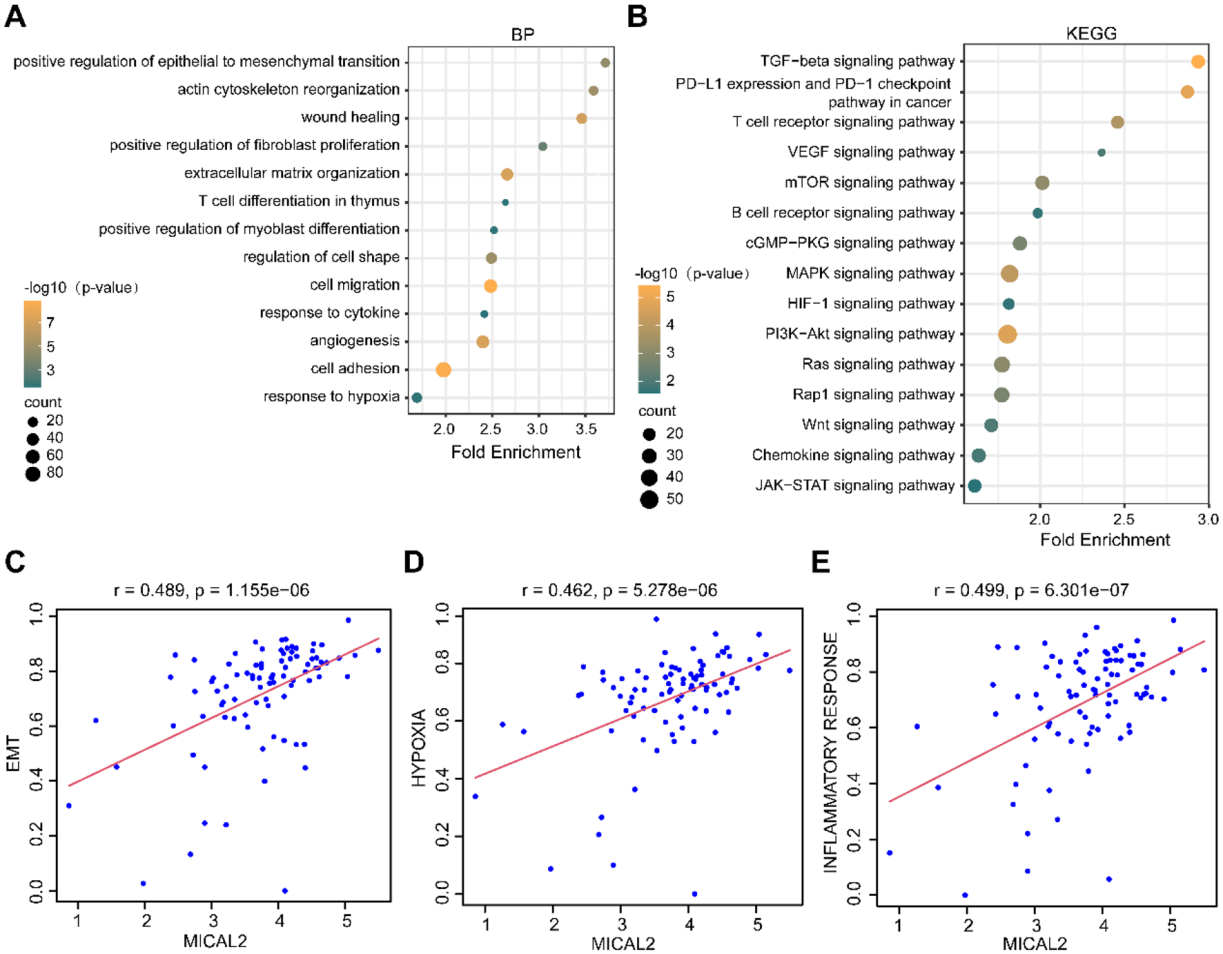
The biological function and pathways of MICAL2 in pancreatic cancer. (**A**) GO analysis and (**B**) KEGG analysis of MICAL2 significantly correlated genes. (**C**–E) Correlation analysis of MICAL2 expression with the scores of EMT, hypoxia, and inflammatory response.

To elucidate the biological function of MICAL2 in pancreatic cancer, we performed GO and KEGG pathway enrichment analyses. The biological process of GO indicated that MICAL2-related genes were mainly involved in stroma-related functions of tumor tissue, such as epithelial to mesenchymal transition (EMT), fibroblast proliferation, myoblast differentiation, extracellular matrix organization, and cell migration, in addition to being enriched in immunity, including T cell differentiation and response to cytokine (Fig. 2A). In terms of pathway enrichment, MICAL2-related genes were mainly associated with stroma and immune-related signaling pathways, such as TGF-β, T cell receptor, and chemokine (Fig. 2B). Further correlation analysis revealed that MICAL2 was positively associated with functional activity scores such as EMT (r = 0.489, p < 0.05, Fig. 2C), hypoxia (r = 0.462, p < 0.05, Fig. 2D), and inflammatory response (r = 0.499, p < 0.05, Fig. 2E).

### MICAL2 is associated with stromal components

**Figure 3:**
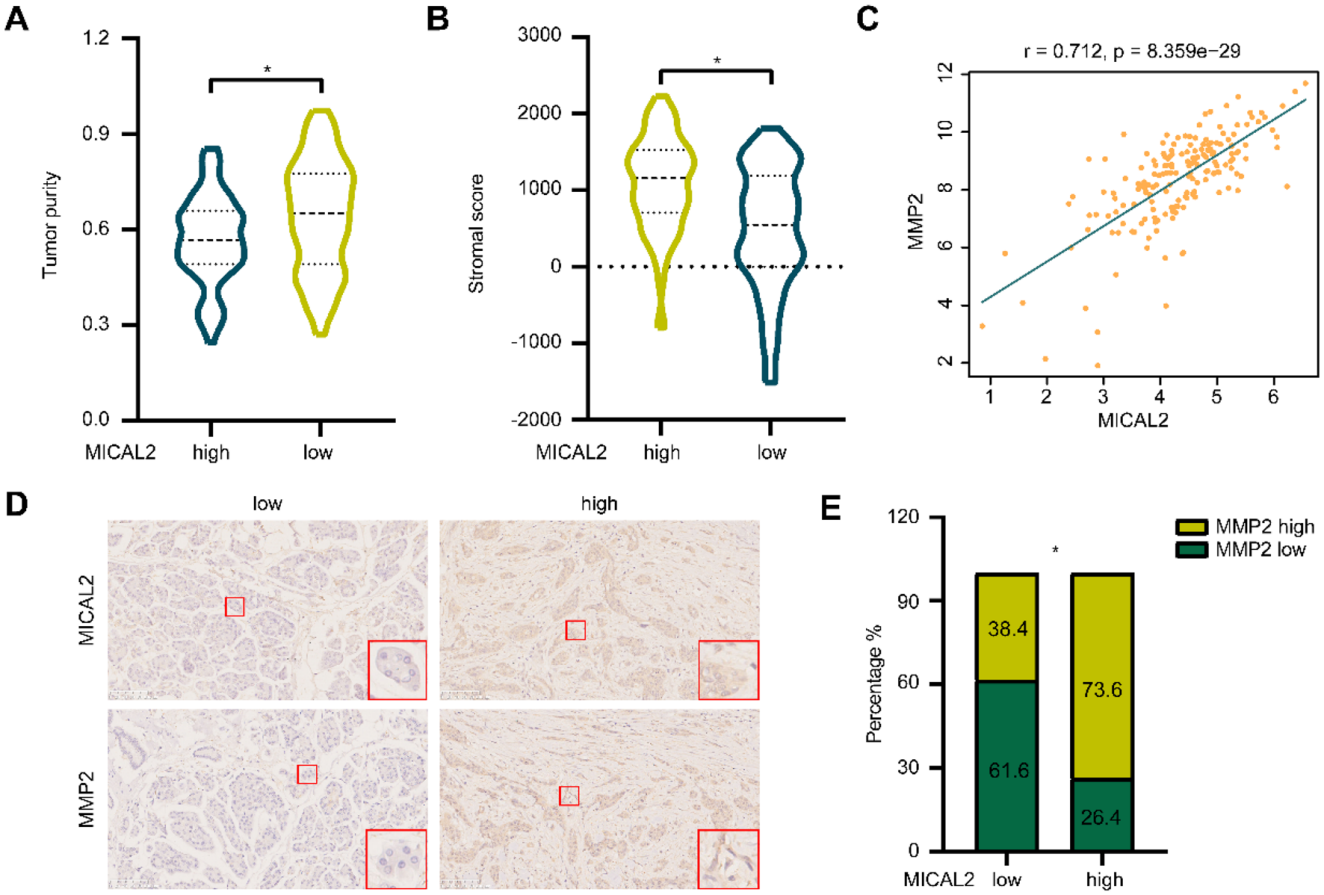
The function of MICAL2 in evaluating the stromal component of pancreatic cancer. (**A**) The relationship between MICLA2 expression and Tumor purity of each pancreatic cancer sample in the TCGA (high MICAL2 expression group: 89, low expression MICAL2 group: 89). (**B**) The relationship between MICLA2 expression and stromal score of each pancreatic cancer sample in the TCGA (high MICAL2 expression group: 89, low expression MICAL2 group: 89). (**C**) Correlation analysis between MICAL2 and MMP2 in the TCGA dataset. (**D**) Representative immunohistochemical images of MICAL2 and MMP2 in pancreatic cancer (bar 100 μm). (**E**) The proportion of MMP2 expression grouped by MICAL2 high/low expression in our cohort (*p < 0.05).

To further determine the specific function of MICAL2 in pancreatic cancer, we calculated the stromal score and tumor purity using the TCGA dataset. The results showed that high MICAL2 expression in pancreatic cancer represented low tumor purity (p < 0.05, Fig. 3A), which indicates higher tumor heterogeneity. However, a higher stromal score was found in the group with high MICAL2 expression (p < 0.05, Fig. 3B), indicating that MICAL2 was closely associated with the stromal components in pancreatic cancer. The tumor stromal components are mainly composed of stromal cells and extracellular matrix. MMP2 can degrade certain components of the extracellular matrix. Therefore, we further analyzed the correlation between MMP2 and MICAL2 in the TCGA dataset. The results of Fig. 3C showed that the expression of MICAL2 and MMP2 were positively correlated (r = 0.712, p < 0.05). The immunohistochemistry staining (Fig. 3D) further confirmed the association between MICAL2 and MMP2. The proportion of patients with high MMP2 expression was increased in the group with high MICAL2 expression (p < 0.05, Fig. 3E).

### MICAL2 is mainly expressed in pancreatic cancer fibroblasts

**Figure 4:**
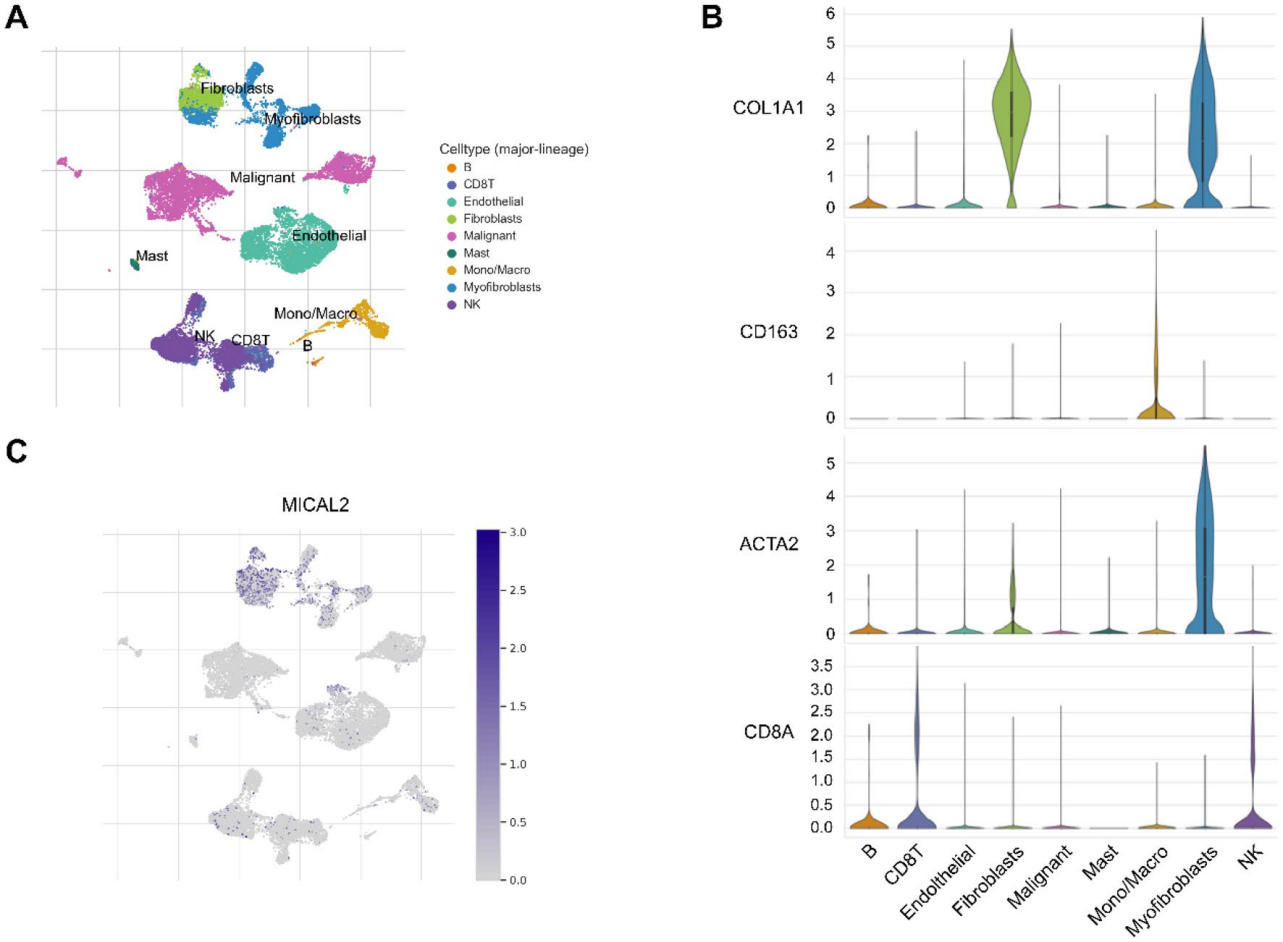
Single-cell transcriptome analysis of MICAL2 in primary pancreatic cancer. (**A**) The two-dimensional plot shows nine cell types in pancreatic cancer. (**B**) The expression of cell biomarkers in different subtypes of cells. (**C**) MICAL2 expression and distribution in single cells based on the above analysis.

To elucidate the cell distribution of MICAL2 expression in pancreatic cancer tissue, we analyzed the single-cell sequencing profile. First, the cells were reduced, clustered, and mapped to a two-dimensional plane. Figure 4A showed nine cell subpopulations, including B cells, CD8 T cells, endothelial, fibroblasts, malignant cells, mast cells, macrophages, myofibroblasts, and natural killer cells. Next, we selected several cell types of interest to demonstrate the expression of biomarkers. Figure 4B showed that the biomarkers were mainly concentrated in the cell types to which they belong, which indicated that cell type annotation was accurate. Finally, based on the above results, we found that MICAL2 was mainly distributed and expressed in fibroblasts (Fig. 4C).

### MICAL2 is positively associated with CAFs-related biomarkers

**Figure 5:**
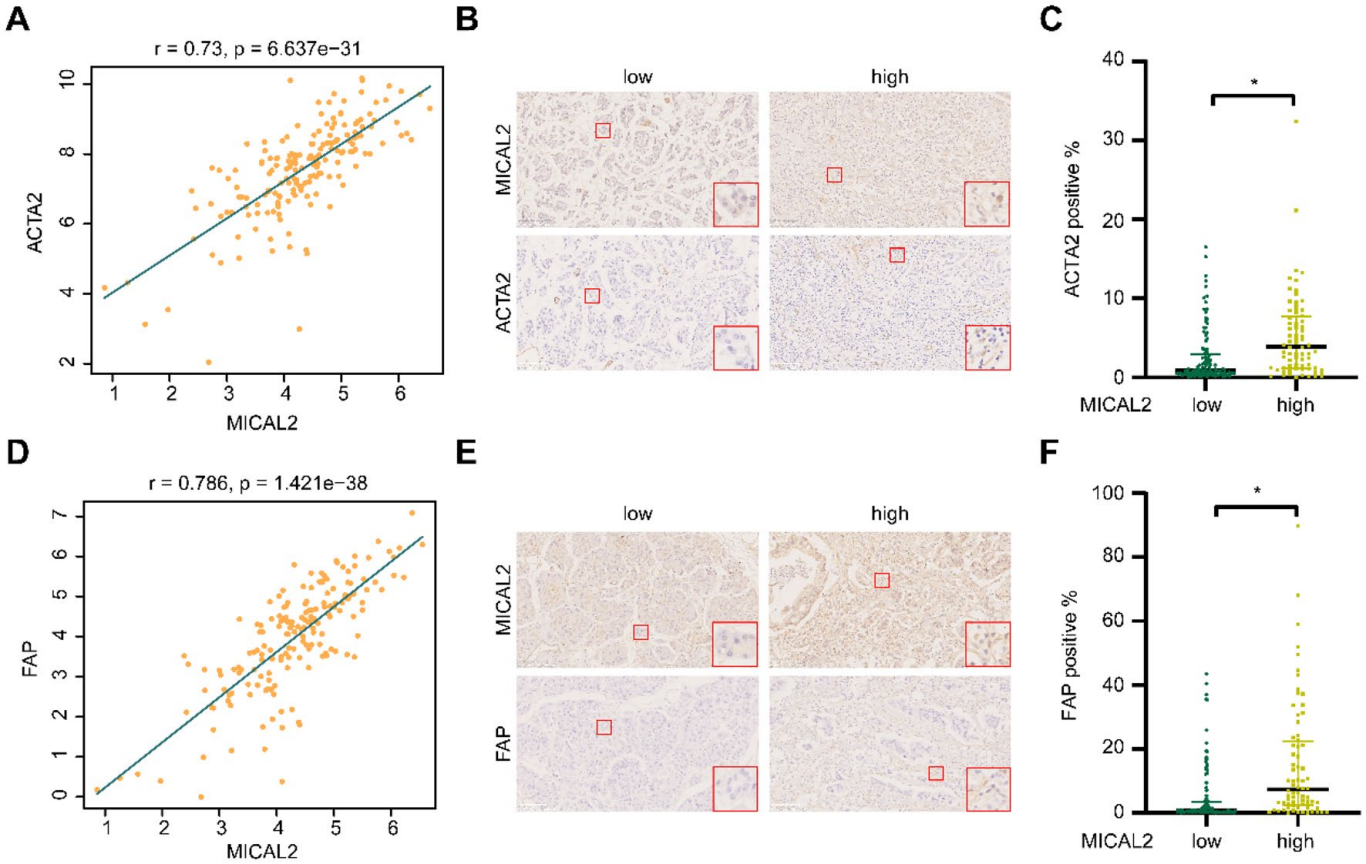
The relationship between MICAL2 and CAFs biomarkers. (**A**) Correlation analysis between MICAL2 and fibroblast biomarker ACTA2 in the TCGA dataset. (**B**) Photographs of immunohistochemical staining of MICAL2 and ACTA2 in pancreatic cancer samples (scale bar 100 μm). (**C**) The differential analysis of ACTA2 in the two groups with high and low MICAL2 expression in our cohort (high MICAL2 expression group: 72, low expression MICAL2 group: 112). (**D**) Correlation analysis between MICAL2 and CAFs biomarker FAP in the TCGA dataset. (**E**) Photographs of immunohistochemical staining of MICAL2 and FAP in pancreatic cancer samples (scale bar 100 μm). (**F**) The difference in FAP expression between the high and low MICAL2 groups in our cohort (high MICAL2 expression group: 72, low expression MICAL2 group: 112) (*p < 0.05).

Further, we also found MICAL2 was positively corrected with CAFs biomarkers, ACTA2 (r = 0.73, p < 0.05, Fig. 5A) and FAP (r = 0.786, p < 0.05, Fig. 5D) in the TCGA dataset. Moreover, we validated the above findings through immunohistochemistry staining of ACTA2 and FAP with pancreatic cancer tissue microarrays. More cells with stronger staining of ACTA2 (Fig. 5B) and FAP (Fig. 5E) were found in a MICAL2^high^ patient with pancreatic cancer compared with a MICAL2^low^ patient. Statistical analysis of all samples staining showed that ACTA2-positive (p < 0.05, Fig. 5C) and FAP-positive (p < 0.05, Fig. 5F) areas significantly increased in the MICAL2^high^ group compared with the MICAL2^low^ group.

### High MICAL2 expression indicates increased macrophage infiltration and decreased CD8 + T cell infiltration

**Figure 6:**
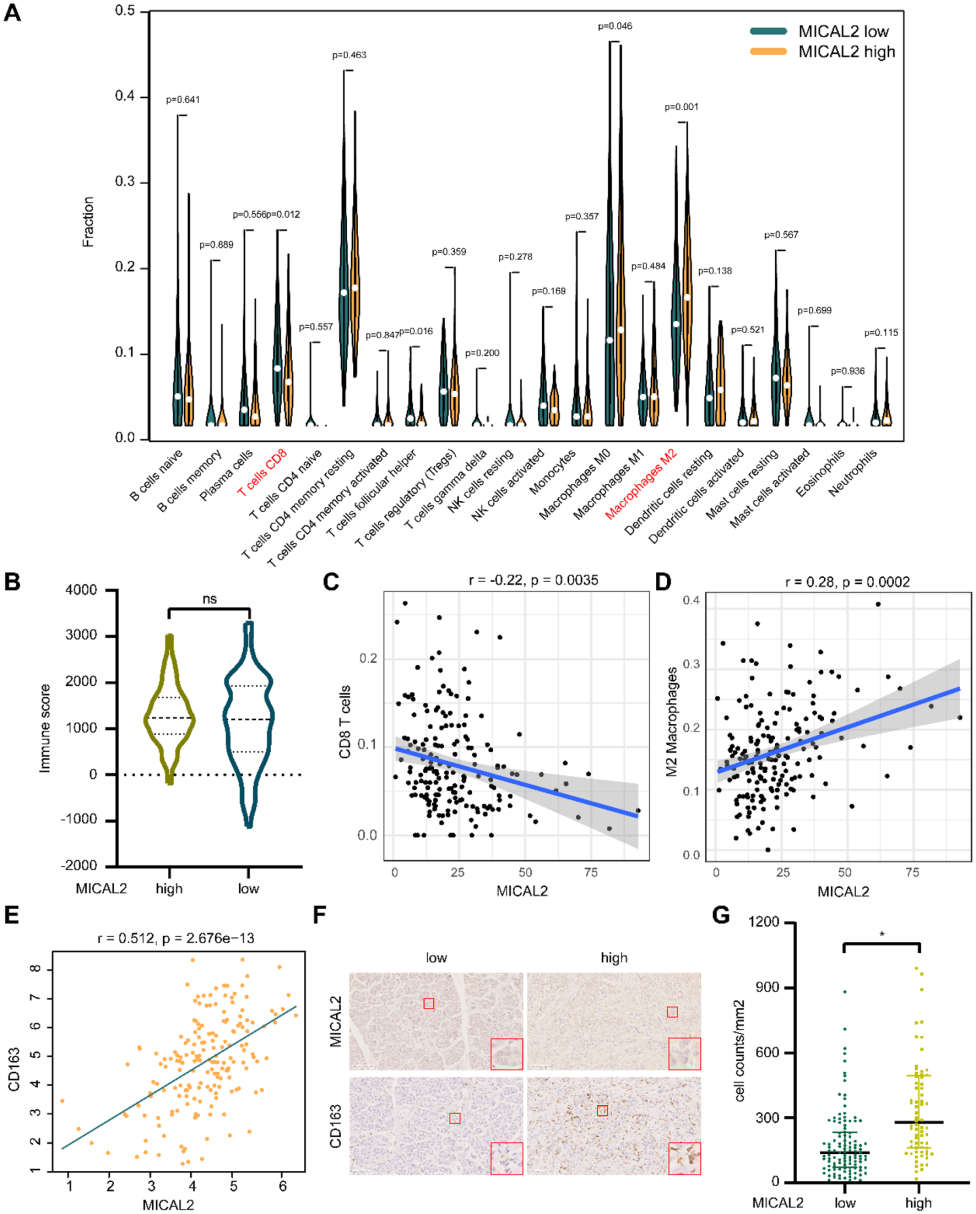
Involvement of MICAL2 in the immune microenvironment of pancreatic cancer. (**A**) Comparison of the infiltration rate of 22 immune cells between patients in the high and low MICAL2 expression group. (**B**) The correlation between MIACL2 expression and immune score (high MICAL2 expression group: 89, low expression MICAL2 group: 89). (**C**) The correlation between MICAL2 expression and CD8 + T cell infiltration. (**D**) The correlation between MICAL2 expression and M2 Macrophage infiltration. (**E**) The correlation analysis between MICAL2 and CD163. (**F**) Representative immunohistochemical images of MICAL2 and CD163 in pancreatic cancer samples (scale bar 100 μm). (**G**) Differential analysis of CD163-positive cell counts grouped by MICAL2 in our cohort (high MICAL2 expression group: 72, low expression MICAL2 group: 112) (*p < 0.05).

To explore the role of MICAL2 in the immune microenvironment of pancreatic cancer, we used the “CIBERSORT” algorithm to calculate the infiltration of 22 immune cells and found that there were notable differences between the CD8 + T cells and M2 macrophages. The more M2 macrophage infiltration and lower CD8 + T cell infiltration in the MICAL2^high^ group compared with the MICAL2^low^ group (Fig. 6A). In addition, the immune score had no significant difference in different MICAL2 groups (Fig. 6B). Further investigation of the role of MICAL2 in the immune microenvironment revealed that high MICAL2 expression was negatively correlated with CD8 + T cells infiltration (r = -0.22, p < 0.05, Fig. 6C), but positively correlated with M2 macrophages infiltration (r = 0.28, p < 0.05, Fig. 6D). A strong correlation was observed between MICAL2 and M2 macrophages biomarker CD163 in the TCGA dataset (r = 0.512, p < 0.05, Fig. 6E), while immunohistochemistry staining showed that more CD163-positive cells were observed in the MICAL2^high^ patient with pancreatic cancer compared with the MICAL2^low^ patient (Fig. 6F). Statistical analysis of all samples staining showed that CD163-positive cell counts significantly increased in the MICAL2^high^ group compared with the MICAL2^low^ group (p < 0.05, Fig. 6G). These findings suggested that high MICAL2 expression indicated increased macrophage infiltration and decreased CD8 + T cells, thus facilitating the formation of an immunosuppressive microenvironment in pancreatic cancer.

### High MICAL2 expression is associated with poor prognosis in patients with pancreatic cancer

**Figure 7:**
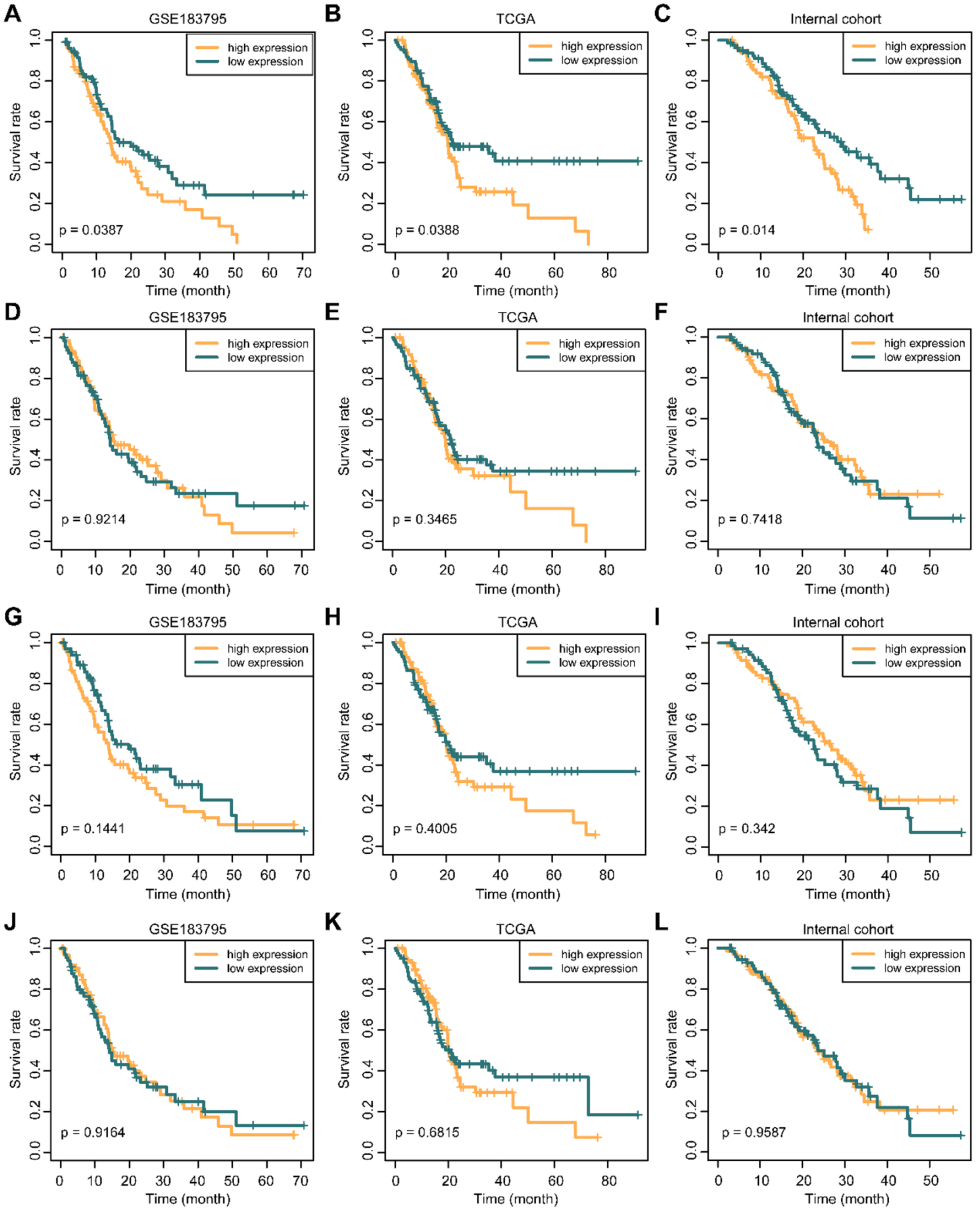
High MICAL2 expression is associated with a poor prognosis. Survival curves of MICAL2 (**A**–**C**), MMP2 (**D**–**F**), ACTA2 (**G**–**I**), and FAP (**J**–**L**) in pancreatic cancer of GSE183795, the TCGA dataset, and our cohort.

We performed a Kaplan–Meier survival analysis using data from GSE183795, the TCGA dataset, and the internal cohort. We divided patients into two groups based on the high/low expression of MICAL2, MMP2, ACTA2, and FAP. The results showed that the survival time of patients in the high MICAL2 expression group was significantly shorter than those of the low MICAL2expression group in GSE183795 (Fig. 7A, p = 0.0387), the TCGA cohort (Fig. 7B, p = 0.0388), and internal cohort (Fig. 7C, p = 0.014). However, the expression levels of MMP2 (Fig. 7D–F), ACTA2 (Fig. 7G–I), and FAP (Fig. 7J–L) were not associated with prognosis in the three cohorts. MICAL2 was associated with the expression of these genes, but not all genes may have prognostic features in biological perspective. It was further illustrated that MICAL2 may act as a prognostic biomarker for pancreatic cancer.

### MICAL2 represents an independent prognostic biomarker for pancreatic cancer

**Figure 8:**
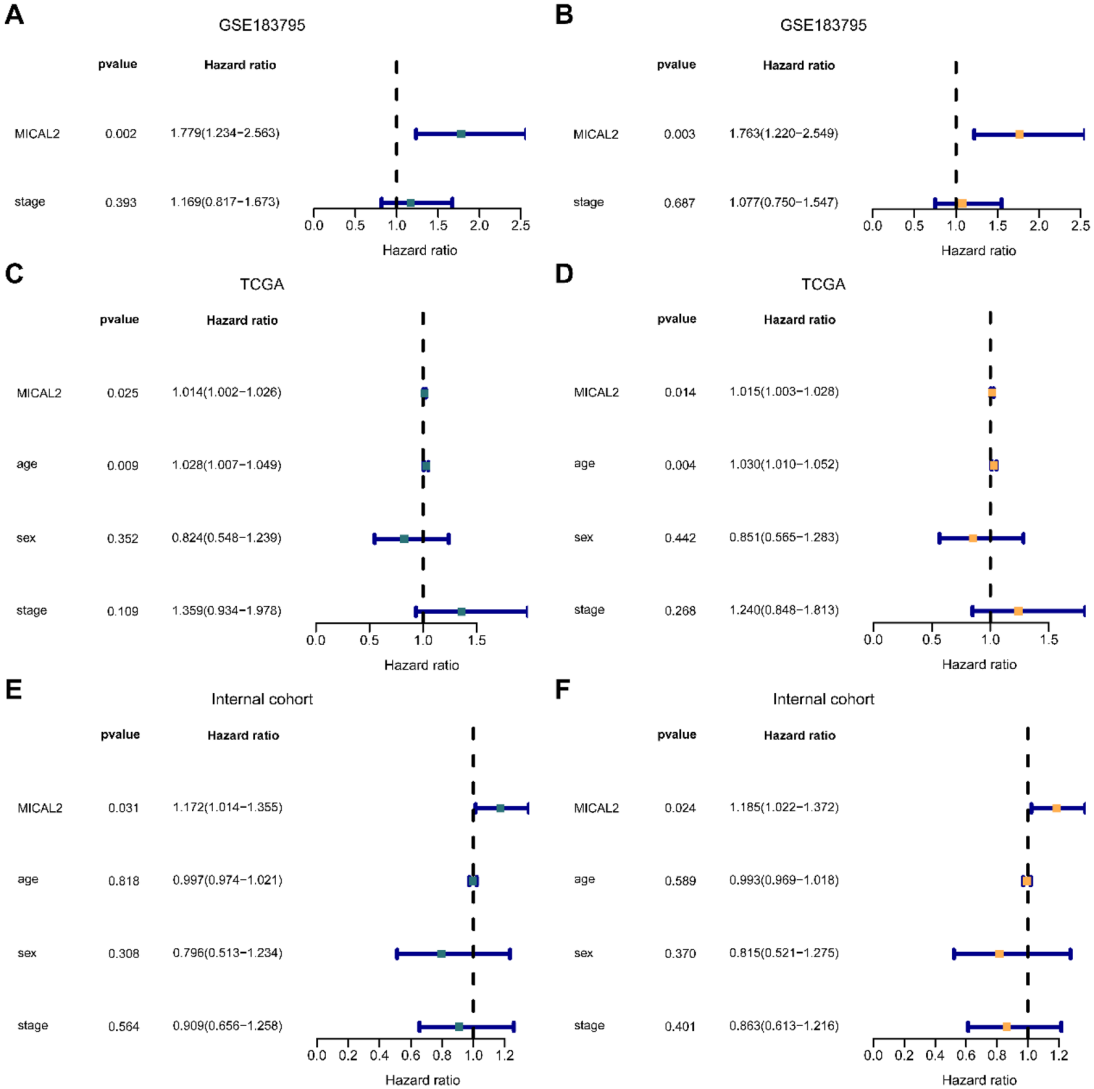
Independent prognostic analysis of MICAL2 in pancreatic cancer. (**A**) Univariable and (**B**) Multivariable Cox regression analysis of MICAL2 expression and other clinical factors in GSE183795. (**C**) Univariable and (**D**) Multivariable Cox regression analysis of MICAL2 expression and other clinical factors in the TCGA dataset. (**E**) Univariable and (**F**) Multivariable Cox regression analysis of MICAL2 expression and other clinical factors in our cohort.

In addition, we performed univariable and multivariable Cox regression analysis to investigate the independent prognostic significance of MICAL2 expression and clinical factors based on three datasets. As shown in Fig. 8, clinical factors were not independent predictors of patient prognosis in the three datasets. However, MICAL2 was an adverse prognostic factor in the Cox regression analysis in GSE183795 [Fig. 8A, univariable hazard ratio (HR): 1.779, 95% CI 1.234–2.563, p = 0.002; Fig. 8B, multivariable HR: 1.763, 95% CI 1.22–2.549, p = 0.003], the TCGA cohort (Fig. 8C, univariable HR: 1.014, 95% CI 1.002–1.026, p = 0.025; Fig. 8D, multivariable HR: 1.015, 95% CI 1.003–1.028, p = 0.014), and internal cohort (Fig. 8E, univariable HR: 1.172, 95% CI 1.014–1.355, p = 0.031; Fig. 8F, multivariable HR: 1.185, 95% CI 1.022–1.372, p = 0.024). These findings indicated that MICAL2 expression was an independent unfavorable prognostic biomarker for pancreatic cancer.

## Discussion

Pancreatic cancer is a highly malignant tumor of the digestive system. A key factor that contributes to the poor prognosis seen in pancreatic cancer is the obscurity of clinical symptoms during its early stages, making early detection difficult. Most patients are diagnosed only during late stages by which time they have spread outside the pancreas, making radical surgical treatment no longer possible^36,37^. Thus, systemic chemotherapy remains the only available primary treatment option. However, the few Food and Drug Administration-approved chemotherapies and targeted therapies that are available have only managed to increase the 5-year overall survival rate for patients with pancreatic cancer from 2%, 10 years ago, to 11%, by 2022^38^. Therefore, developing new therapeutics is imperative for clinical treatment and success.

In recent years, MICAL2 has been identified as a novel pro-tumorigenic factor^39^. Previous studies have indicated that, in addition to being highly expressed in cancers, MICAL2 is associated with poor prognosis^23,24,40,41^. In our study, we found that MICAL2 was highly expressed in pancreatic cancer samples. Survival analyses have revealed that high MICAL2 expression levels were significantly associated with poor survival rates in patients with pancreatic cancer. Furthermore, the ROC curve indicated that MICAL2 shows potential diagnostic capability for pancreatic cancer. These findings indicated that a significant increase in MICAL2 expression may be considered a potential diagnostic and prognostic biomarker in pancreatic cancer.

MICAL2 acts as a novel gene that regulates EMT, a process involved in cancer growth and invasion^19^. In pancreatic cancer, GO enrichment analysis revealed that MICAL2 is mainly enriched in EMT, ECM organization, and other biological functions related to tumor metastasis. Furthermore, high MICAL2 expression causes an increase in the stromal components in pancreatic cancer samples. Matrix metalloproteinases are a family of zinc-dependent ECM remodeling endopeptidases that can degrade almost every component of the ECM^42^. MMP2, a member of the Matrix metalloproteinases family, was positively correlated with MICAL2, confirming that MICAL2 shows potential for promoting tumor metastasis by degrading the ECM. Reportedly, MICAL2 expression in the neoangiogenic endothelium of gastric, renal, breast, glioblastoma, and cardiac mucinous tumors was closely correlated with angiogenic activity resulting from the inflammatory response^43^. GO enrichment analysis revealed that MICAL2 was associated with angiogenesis and inflammatory responses.

CAFs are strongly associated with changes in the stromal content of pancreatic cancers^44^. Single-cell analysis revealed that MICAL2 was mainly concentrated in the fibroblasts of pancreatic cancer, while immunohistochemical staining indicated that MICAL2 expression was positively correlated with the expression of CAFs biomarkers, FAP and ACTA2, proving that MICAL2 was closely associated with CAFs in pancreatic cancer. Although the association between MICAL2 and CAFs remains unexplored, some studies have investigated the potential functions of MICAL2 and the mechanisms underlying them and found that MICAL2 expression was elevated in epidural fibrotic tissues and TGF-β1-stimulated fibroblasts^45^, thereby revealing that high MICAL2 expression may cause an increase in stromal cells, including CAFs.

More studies have found that CAFs play an important role in the formation of immunosuppressive microenvironment of tumors, where they interact with tumor-infiltrating immune cells and other immune components by secreting a variety of cytokines, growth factors, chemokines, exosomes, and other effector molecules that allows cancer cells to evade immune surveillance. One specific study found that immunosuppression by fibroblasts may also involve the polarization of M0 macrophages to M2 macrophages, which promotes tumor development^46^. It was also found that CAFs induced an increase in the expression of programmed cell death protein 1 on the surface of M2 macrophages, which further increased their immunosuppressive capacity^47^. In addition to interacting with macrophages, CAFs may also cause a reduction in CD8 + T cell infiltration. For example, it may reduce CD8 + T cell aggregation and inhibit their cytotoxicity to tumor cells by secreting IL-6 and TGF-β^48^. Moreover, CAFs may also block the migration of CD8 + T cells to the tumor site by releasing VEGF to reduce cell adhesion molecules or mediate ECM modifications leading to stromal densification^49,50^. These studies substantiate our findings which indicated that MICAL2, expressed in fibroblasts, may induce polarization of M2 macrophages and decrease CD8 + T cell infiltration.

However, the interaction between CAFs and immune cells mediated by MICAL2 has not been further explored in this study. Future research should be directed at gaining a better understanding of the complex mechanism underlying the role played by MICAL2 in promoting the interaction between CAFs and immune cells, as this may enable effective targeted therapy and immunotherapy to be developed against pancreatic cancer.

In conclusion, our study revealed that MICAL2, mainly expressed in fibroblasts, is an independent and adverse prognostic factor and facilitates metastasis and the formation of immunosuppressive microenvironment in pancreatic cancer. These findings may help provide clues for diagnosis, targeted therapy combination immunotherapy, and prognosis for pancreatic cancer.

### Supplementary Information

Supplementary Table 1.

## Data Availability

All data in this study were included in this published article [and its supplementary information files].

## Abbreviations

MICAL2: Microtubule associated monooxygenase, calponin and LIM domain containing 2
CAFs: Cancer-associated fibroblasts
TME: Tumor microenvironment
VEGF: Vascular endothelial growth factor
IL-6: Interleukin-6
ECM: Extracellular matrix
CXCL9: C-X-C motif chemokine ligand 9
TGF-β: Transforming growth factor-β
GEO: Gene expression omnibus
TCGA: The cancer genome atlas
GO: Gene ontology
KEGG: Kyoto encyclopedia of genes and genomes
FAP: Fibroblast activation protein alpha
ACTA2: Actin alpha 2
MMP2: Matrix metalloproteinase 2
ROC: Receiver operating characteristic
CI: Confidence interval
EMT: Epithelial to mesenchymal transition
HR: Hazard ratio
