# Supplementary material for: MICAL2 implies immunosuppressive features and acts as an independent and adverse prognostic biomarker in pancreatic cancer
Source: Sci Rep. 2024 Feb 7;14:3177. doi: 10.1038/s41598-024-52729-6 (PMC10850094; doi:10.1038/s41598-024-52729-6)
Supplement: Supplementary file 1 — Supplementary Table 1. [file 41598_2024_52729_MOESM1_ESM.docx]

Supplementary Table 1. Cohorts collected in the study.

| Clinical factors | GSE16515  (n = 36) | GSE183795  (n = 139) | TCGA  (n = 178) | Internal cohort  (n = 184) |
| --- | --- | --- | --- | --- |
| Age | <= 60  (11, 30.6%)  > 60  (25, 69.4 %) | <= 60  -  > 60  - | <= 60  (59, 33.1 %)  > 60  (119, 66.9 %) | <= 60  (76, 41.3 %)  > 60  (108, 58.7 %) |
| Sex | Male  (22, 61.1 %)  Female  (14, 38.9 %) | Male  -  Female  - | Male  (98, 55.1 %)  Female  (80, 44.9 %) | Male  (109, 59.2 %)  Female  (75, 40.8 %) |
| Stage | I  -  II  -  III  -  IV  - | I  (8, 5.8 %)  II  (104, 74.8 %)  III  (19, 13.7 %)  IV  (6, 4.3 %)  Unknown  (2, 1.4 %) | I  (22, 12.4 %)  II  (149, 83.7 %)  III  (3, 1.7 %)  IV  (4, 2.2 %) | I  (19, 10.3 %)  II  (113, 61.4 %)  III  (23, 12.5 %)  IV  (14, 7.6 %)  Unknown  (15, 8.2 %) |

GEO: Gene Expression Omnibus database TGCA: The Cancer Genome Atlas database
